# Adjuvant Immunotherapy in Curative Intent Esophageal Cancer Resection Patients: Real-World Experience within an Integrated Health System

**DOI:** 10.3390/cancers15225317

**Published:** 2023-11-07

**Authors:** Hyunjee V. Kwak, Kian C. Banks, Yun-Yi Hung, Nathan J. Alcasid, Cynthia J. Susai, Ashish Patel, Simon Ashiku, Jeffrey B. Velotta

**Affiliations:** 1 Department of Surgery, University of California, San Francisco-East Bay, 1411 E 31st Street, QIC 22134, Oakland, CA 94602, USA; kibanks@alamedahealthsystem.org (K.C.B.); nalcasid@alamedahealthsystem.org (N.J.A.); csusai@alamedahealthsystem.org (C.J.S.); 2 Biostatistical Consulting Unit, Division of Research, Kaiser Permanente Northern California, 2000 Broadway, Oakland, CA 94612, USA; yun-yi.hung@kp.org; 3 Division of Thoracic Surgery, Department of Surgery, Kaiser Permanente Northern California, 3600 Broadway, Oakland, CA 94612, USA; ashish.r.patel@kp.org (A.P.); simon.k.ashiku@kp.org (S.A.); jeffrey.b.velotta@kp.org (J.B.V.); 4 Department of Surgery, University of California, San Francisco School of Medicine, 533 Parnassus Ave, San Francisco, CA 94143, USA; 5 Department of Clinical Medicine, Kaiser Permanente Bernard J. Tyson School of Medicine, 98 S Los Robles Ave, Pasadena, CA 91101, USA

**Keywords:** esophageal cancer, immunotherapy, nivolumab

## Abstract

**Simple Summary:**

This is an invited study for the special edition, “Role of Immunotherapy in Gastroesophageal Cancers: Advances, Challenges and Future Strategies”. Esophageal cancer is a disease with one of the lowest survival rates. For patients who have resectable tumors, current guidelines recommend treatment with neoadjuvant chemoradiation followed by surgery if feasible. Recent clinical trials have suggested that treating patients with residual pathologic disease with adjuvant immunotherapy can increase overall survival. The aim of our retrospective study was to assess the real-world outcomes of patients at a regionalized esophageal cancer center in a large integrated health system. We found that the majority of patients are unable to complete immunotherapy treatment (82%), most commonly due to disease progression or side effects. In logistic regression, immunotherapy also did not significantly impact 1-year overall survival. Our experience within the first two years of adjuvant immunotherapy being introduced suggests that real-world outcomes vary greatly outside of those seen in a clinical trial.

**Abstract:**

Background: Adjuvant immunotherapy has been shown in clinical trials to prolong the survival of patients with esophageal cancer. We report our initial experience with immunotherapy within an integrated health system. Methods: A retrospective cohort study was performed reviewing patients undergoing minimally invasive esophagectomy at our institution between 2017 and 2021. The immunotherapy cohort was assessed for completion of treatment, adverse effects, and disease progression, with emphasis on patients who received surgery in 2021 and their eligibility to receive nivolumab. Results: There were 39 patients who received immunotherapy and 137 patients who did not. In logistic regression, immunotherapy was not found to have a statistically significant impact on 1-year overall survival after adjusting for age and receipt of adjuvant chemoradiation. Only seven patients out of 39 who received immunotherapy successfully completed treatment (18%), with the majority failing therapy due to disease progression or side effects. Of the 17 patients eligible for nivolumab, 13 patients received it (76.4%), and three patients completed a full course of treatment. Conclusions: Despite promising findings of adjuvant immunotherapy improving the survival of patients with esophageal cancer, real-life practice varies greatly from clinical trials. We found that the majority of patients were unable to complete immunotherapy regimens with no improvement in overall 1-year survival.

## 1. Introduction

Despite ongoing therapeutic innovations, esophageal cancer remains a disease with one of the poorest survival rates, with an overall 5-year survival of 20% [1]. In the United States, the incidence of esophageal adenocarcinoma specifically has increased over the last 15 years [2]. However, on a global scale, esophageal squamous carcinoma continues to dominate, with overall esophageal cancer incidence and mortality increasing to an estimated 957,000 and 880,000 cases, respectively, in 2040 [3]. The current standard of care for resectable, locally advanced esophageal cancer includes neoadjuvant chemoradiation followed by surgery [4,5]. Recent clinical trials have demonstrated the role of immunotherapy in further improving patient survival [6,7]. However, these therapies are often not completed in their entirety in real-life practice due to a variety of reasons, including intolerance to drug side effects, progression of the disease, or patient personal factors prohibiting full follow-up. In addition, as adjuvant immunotherapy has only recently been approved by the Food and Drug Administration (FDA), many centers have not established standardized guidelines for its use. To the authors’ knowledge, we report the first and only initial real-world experience with the use of adjuvant immunotherapy after minimally invasive esophagectomy for esophageal cancer within our integrated health system.

## 2. Materials and Methods

A retrospective cohort study was conducted using data from the Kaiser Permanente Northern California (KPNC) health system, a nonprofit, integrated network of facilities that provide care to approximately 50% of the region’s insured population with nearly 5 million members. Cancer care, specifically esophageal and gastroesophageal cancer, is centralized within the system, with the regional thoracic surgery center of excellence being Kaiser Permanente Oakland Medical Center (KP-Oakland). All adult patients undergoing minimally invasive Ivor-Lewis esophagectomy at KP-Oakland between 1 September 2017 and 15 November 2021 were included. No patients underwent open or hybrid operations. After receiving neoadjuvant treatment, patients were restaged within 4 weeks by PET scan. If there was no evidence of metastases, patients proceeded to surgery. Five patients with multiple cancers were excluded. Baseline clinicopathologic characteristics including year of diagnosis and surgery, age, sex, race/ethnicity, body mass index (BMI), smoking history, alcohol abuse, Charlson comorbidity index (CCI), operative duration, histology, adjuvant chemoradiation, neoadjuvant chemoradiation, and 90-day and 1-year mortality were extracted from KPNC electronic medical records and confirmed by chart review. The primary outcome was death due to all causes 1 year after surgery, and multivariable logistic regression analysis was performed to identify factors that impacted overall survival. The regression model was adjusted for age, receipt of immunotherapy, and receipt of adjuvant chemoradiation. Patients who received immunotherapy were further chart reviewed for the number of doses they received, completion of therapy, reason for stopping treatment, and specific side effects. Patients who received surgery in 2021 were also reviewed to determine their eligibility to receive nivolumab, which became FDA-approved at that time, actual receipt of treatment, and completion of a full course of treatment. Eligibility criteria were based on those of patients included in the CheckMate 577 trial [6]. A subanalysis was performed for patients with pathologic stage II and III disease who received surgery in 2021. The same clinicopathologic characteristics listed above, as well as R0 resection rate, lymph node yield, and disease-free survival, were compared between those patients who received immunotherapy and those who did not. Normally distributed continuous variables were summarized as mean and standard deviation and compared using two sample t tests. Chi-square tests or Fisher exact tests were performed for categorical variables. All analyses were performed using SAS 9.4 (SAS Institute, Cary, NC, USA). *P*-values were 2-sided, with a significance threshold of 0.05.

## 3. Results

A total of 181 patients were identified during the study period. After excluding five patients with multiple cancers, a total of 39 patients received adjuvant immunotherapy, and 137 did not (Table 1). The study population was predominantly white and male. There were no significant differences between patients who received immunotherapy compared to those who did not with respect to age, sex, race and ethnicity, BMI, smoking history, alcohol abuse, CCI, neoadjuvant therapy, operative duration, or histology. Significant differences between the two groups were found for year of diagnosis and surgery, stage, and adjuvant therapy in which significantly more patients after minimally invasive esophagectomy received adjuvant immunotherapy later the year of surgery as well as diagnosis. There were no statistically significant differences in 90-day or 1-year mortality between the groups (Table 1). The multivariable logistic regression analysis showed that immunotherapy was not found to have a statistically significant impact on 1-year survival after adjusting for age, receipt of immunotherapy, and receipt of adjuvant chemoradiation (Table 2).

**Figure 1:**
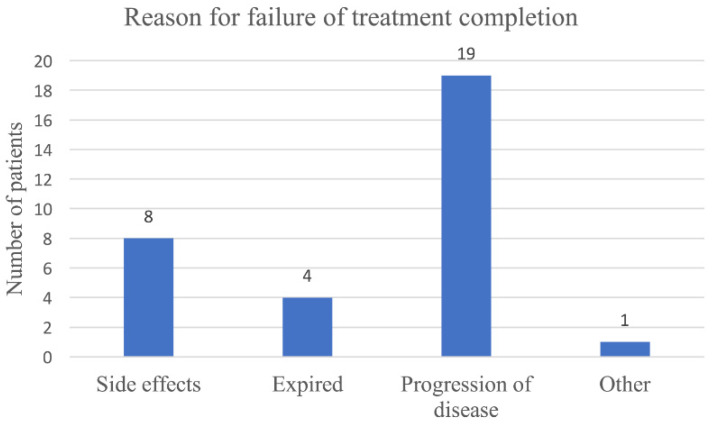
Reasons why 32 patients failed to complete immunotherapy treatment.

Table 3 summarizes the number and type of adverse events, dosing frequency, and treatment completion by type of immunotherapy. Of the 39 patients who received immunotherapy, 19 received nivolumab, 17 received pembrolizumab, 11 received trastuzumab, and two received ipilimumab. Of the 39 patients, nine patients received a multidrug regimen (Table 3). Of the 39 patients, 32 (82%) did not complete therapy, and seven patients successfully completed therapy (18%). The reasons for failure of treatment completion are displayed in Figure 1. Eight patients terminated therapy due to side effects (25%), most commonly rash or fatigue. An additional four patients expired during treatment, 19 patients stopped therapy due to disease progression, and one patient stopped treatment due to a change in their hormone receptor status.

A review of all 30 patients who received surgery in 2021 after nivolumab was FDA-approved showed that four out of 17 patients who were eligible for adjuvant nivolumab did not receive the treatment (23.5%). Two eligible patients were not offered the therapy, which may reflect the difficulty in standardizing practice patterns even within an integrated health system in the wake of new data. Two more eligible patients declined or expired prior to meeting with the medical oncologist. Of the 13 patients who were qualified to receive nivolumab and proceeded with treatment, three patients successfully completed treatment (23.1%). Of the 10 patients who were unable to complete the full year of adjuvant treatment, six were unable to continue due to the progression of the disease (60%). The remaining four patients (40%) stopped therapy due to side effects, most commonly colitis.

A subanalysis was performed on just patients with pathologic stage II and III disease who received surgery in 2021 to better emulate the patient cohort in the CheckMate 577 trial. There were 10 patients who received immunotherapy and six who did not (Table 4). There were no differences in baseline clinicopathologic characteristics, including age, sex, race/ethnicity, BMI, smoking history, alcohol abuse, CCI, operative duration, histology, adjuvant treatment, or pathologic stage between the two groups. There were also no statistical differences in R0 resection rate, lymph node yield, 30-day overall survival, 1-year overall survival, or disease-free survival between the two groups. Median disease-free survival for our immunotherapy patients was 11.5 months compared to the 22 months seen in CheckMate 577 [6].

## 4. Discussion

We sought to review our integrated health system’s initial use of adjuvant immunotherapy in resected esophageal cancer to establish the first real-world outcomes in a large community setting. As expected, of the 176 patients in our 4-year study period, only 39 patients received immunotherapy, highlighting the relative novelty of this treatment. Between the two patient groups, patients diagnosed and operated on in later years and those patients with higher stages were more likely to receive immunotherapy per the changing treatment guidelines. In multivariable logistic regression analysis, immunotherapy was not found to be statistically significant in affecting 1-year mortality after adjusting for age and receipt of adjuvant chemoradiation. We acknowledge that a one-year follow-up period is short, and further studies are required to accurately comment on the effect of immunotherapy on survival in our patients.

Our tertiary referral center within an integrated health system is unique in that it is the regional thoracic surgery center of excellence, requiring the presence of fellowship-trained thoracic surgeons, medical and radiation oncologists, interventional radiologists, and advanced endoscopists. Referral to a center of excellence is expedited through a standardized tagging system based on pathology diagnoses of esophageal or gastroesophageal cancer diagnoses as previously described [8]. The electronic medical record system then automatically sends the pathology report with the corresponding patient medical record number to the inboxes of thoracic surgeons and medical oncologists at the center of excellence for review. Multidisciplinary tumor boards are held weekly to care for patients found to have esophageal and gastroesophageal junction cancer. As such, patients are able to establish care with specialists faster and are more likely to have superior short- and long-term outcomes in esophageal cancer as well as in other types of cancer [9,10,11,12].

Overall, our institution’s medical oncologists were able to quickly integrate the findings of the CheckMate 577 trial, as evidenced by 13 out of 17 patients eligible for nivolumab in 2021 receiving therapy. Forty percent of the patients were discontinued on nivolumab due to its well-documented autoimmune side effects [6,13,14]. In the CheckMate 577 trial, 34% of patients receiving nivolumab had a grade 3 or 4 adverse event, resulting in 9% of patients discontinuing therapy [6]. Our small sample size likely affects this discrepancy in the proportion of treatment discontinuation compared to CheckMate 577. Our institution’s medical oncologists consider a 1-year duration of nivolumab to be a complete course. Of note, in the CheckMate 577 trial, the median duration of nivolumab administration was 10.1 months in 532 patients [6]. Of the patients receiving nivolumab at our institution, 23.1% successfully completed 1 year of therapy, suggesting that a prolonged course of nivolumab could potentially be completed in the right patient population. To better compare our experience with that of CheckMate 577, we looked at the median disease-free survival of our patients with pathologic stage II and III disease who received surgery in 2021. Survival in our immunotherapy cohort was shorter than seen in CheckMate 577 (11.5 vs. 22 months), highlighting the difference in real-world outcomes compared to those within a clinical trial. Future studies with more patients and a longer follow-up duration are required for a more detailed analysis.

Despite being a center of excellence within an integrated health system that centralizes cancer care and incorporates new therapies into practice in a timely fashion, we found that the majority of our patients are unable to finish immunotherapy (of any type, not just nivolumab) in its entirety for the full year. Of the patients who were unable to complete treatment, the majority were due to disease progression. However, adverse effects were the cause of 20% of patients stopping treatment, the most common being rash, fatigue, or weight loss. Several studies have shown that the impact of adverse effects reported by participants in clinical trials is understated for both molecular and immunotherapies [15,16,17,18,19]. A systematic review by Peron and colleagues showed that 90% of 325 randomized controlled trials (RCTs) did not adhere to the Consolidated Standards of Reporting Trials (CONSORT) guidelines in the reporting methodology of adverse event collection [16]. In addition, descriptions of adverse events leading to withdrawal were not reported in 85% of RCTs, and 62% of RCTs failed to attribute the adverse events to the trial interventions [16]. This raises the concern that clinical trials do not report the majority of adverse events. With respect to immunotherapy specifically, a systematic review by Chen and colleagues of 50 trials showed that 60% of trials did not describe adverse event collection methodology, and 48% of trials did not report data on patient withdrawal due to adverse events [18]. Taken together, the adverse events reported in clinical trials appear to be greatly understated despite established guidelines. The data from clinical trials on adverse events must be used cautiously when applied to real-world adherence to an immunotherapy regimen, especially in the case of our patient population who underwent not only neoadjuvant chemoradiation but minimally invasive esophagectomy prior to their institution of adjuvant immunotherapy. Many previous studies have shown the significant delay to adjuvant systemic therapy after esophagectomy regardless of minimally invasive versus open approach and that postoperative complications and adverse events from the therapies themselves negatively affect short- and long-term survival [20,21,22,23]. Our findings, in addition to the previous literature, further highlight the inherent difficulties that esophageal cancer patients specifically are faced with in regard to tolerance to undergo further adjuvant treatment. Adherence to adjuvant treatment will vary greatly by institutional experience and the corresponding unique patient populations.

An additional important point regarding the generalizability of the results of clinical trials to the general patient population is the lack of diversity of the participants. Clinical trial enrollment has numerous disparities, including gender, age, race/ethnicity, and medical comorbidity burden [24,25,26,27,28]. Murthy and colleagues reported that racial and ethnic minorities and the elderly were less likely to participate in cancer trials [24]. Our patient cohort is predominantly white and male, similar to the patients who are recruited at specialized cancer centers for treatment in clinical trials, including CheckMate 577. However, 40.1% of our immunotherapy patients are non-white, with 5.1% being Black and 10.3% being Hispanic (Table 1). This is consistent with or greater than the proportion of Black and Hispanic patients currently reported to participate in cancer clinical trials, 4–6% and 3–6%, respectively [24,27,28]. Furthermore, two-thirds of our patients receiving immunotherapy had a high number of comorbidities with a Charlson Comorbidity Index of 7+ (Table 1). This is significant in the context of research published by Unger and colleagues, which reported that the presence of 1 or more comorbidities resulted in decreased discussion of clinical trials (from 44.1% to 37.2%), offers of clinical trials (from 21.7% to 15.7%), and participation in clinical trials (from 11.3% to 7.8%) [25]. Lastly, our patient population also varied in extremes of age, ranging from the 2nd to the 8th decades of life (Table 1). Overall, our population is more diverse than those typically seen in clinical trials with respect to race and ethnicity, age, and comorbidity burden. Ongoing follow-up is currently underway to assess our institution’s long-term outcomes and whether they are comparable to those of CheckMate 577.

The landscape of esophageal cancer treatment is ever-changing. Prior to the CheckMate 577 trial, the FLOT4 trial was published in 2019, which showed that neoadjuvant plus adjuvant docetaxel-based chemotherapy had improved survival compared to non-docetaxel-based regimens [29]. Of note, this trial did not include patients with esophageal cancer (only locally advanced, resectable gastric and gastroesophageal cancers). However, the FLOT4 regimen has increasingly been adopted for lower esophageal cancers as an alternative to neoadjuvant chemoradiation recommended by the CROSS trial. Several randomized clinical trials are currently enrolling or have preliminary data showing that survival outcomes are similar between FLOT4 and CROSS regimens [30,31,32]. However, as the CheckMate 577 trial did not include any patients who had only received neoadjuvant chemotherapy, patients treated with the FLOT4 regimen are not eligible for adjuvant nivolumab. The EORTC VESTIGE trial aimed to combine adjuvant immunotherapy with patients who received neoadjuvant chemotherapy [33,34]. The trial enrolled patients with locally advanced resectable gastroesophageal junction cancers who received neoadjuvant chemotherapy and were randomized to adjuvant nivolumab plus ipilimumab versus chemotherapy (same as neoadjuvant regimen) after surgery. Enrollment was discontinued early in June 2022 after data from 191 of a planned 240 patients were reviewed. These results showed that adjuvant nivolumab + ipilimumab did not improve disease-free survival compared to chemotherapy. Future studies are needed for patients who were treated with neoadjuvant chemotherapy only and their outcomes after receiving adjuvant nivolumab.

There are several other clinical trials involving immunotherapy for esophageal cancer [35,36]. For patients with locally advanced unresectable disease, the KEYNOTE-975 trial will compare first-line pembrolizumab plus definitive chemoradiation compared to chemoradiation alone [35]. The trial is currently enrolling with a goal of 600 patients by February 2026. In the NATION-2203 trial, nivolumab (or placebo) will be given in the neoadjuvant setting in combination with chemotherapy for patients with resectable esophageal cancer [36]. After surgery, the patients who have not achieved complete pathological response will be given adjuvant nivolumab for up to 1 year. The future results of these trials will supplement those of CheckMate 577 and greatly expand our understanding of the role of immunotherapy in patients with esophageal cancer.

Strengths of our study include KPNC’s comprehensive electronic medical record system and the inclusion of multiple facilities referring patients to our thoracic surgery center of excellence. The inclusion of multiple hospitals across Northern California resulted in a more diverse patient population than the average population seen in clinical trials [37]. In addition, our integrated health system is distinctive in its centralization of cancer care, decreasing the amount of time it takes for the incorporation of newly approved treatments into practice as well as improving both short-term and long-term survival outcomes [9,10,11,12]. Thus, our study potentially, more than others, can possess adequate patient volumes to study more recent novel therapeutic agents such as adjuvant nivolumab after minimally invasive esophagectomy.

There are multiple limitations of this study, including its retrospective nature and smaller overall sample size. However, we have included nearly 40 patients in this study who have received neoadjuvant chemoradiation followed by minimally invasive esophagectomy and adjuvant immunotherapy in a real-world community setting, which is the largest data source to date in the literature. In the future, with further adoption of adjuvant nivolumab after resection for esophageal cancer via the CheckMate 577 protocol around the world, more in-depth, robust, real-world prospective data can be produced. As nivolumab only became approved in mid-2021, our follow-up time is also limited, with too many confounding factors to make short-term (90-day and 1-year mortality) outcome data significant. Future follow-up is underway to investigate median disease-free survival in the adjuvant nivolumab group at our institution to further verify the improved survival seen in CheckMate 577 in a real-world community setting as our current disease-free survival of 11.5 months is much shorter than the 22 months seen in CheckMate 577. Future analyses are required to further detail long-term survival and outcomes in this patient population.

## 5. Conclusions

Adjuvant immunotherapy has been shown to have an encouraging impact on patient survival in resectable esophageal cancer in the clinical trial setting [6]. However, outside of a clinical trial, there are numerous barriers to successful treatment in a real-world setting. At our regionalized center of excellence with a diverse patient population, we found that most patients are unable to complete immunotherapy treatment due to disease progression or side effects, and immunotherapy was not found to have a statistically significant impact on 1-year survival. Although the incorporation of new practice standards was done in a timely fashion, as evidenced by the majority of patients eligible for nivolumab receiving the therapy, completion of treatment was still a problem for patients. Further work is required to tailor immunotherapy delivery and adherence in diverse patient populations.

## Figures and Tables

**Table 1:** Patient demographics and mortality by receipt of immunotherapy.

Variables	Total (N = 176)	on Immunotherapy (N = 39)	Not on Immunotherapy (N = 137)	*p* Value
Year of cancer diagnosis				<0.001 ^‡^
2016	4 (2.3)	0	4 (2.9)	
2017	26 (14.8)	6 (15.4)	20 (14.6)	
2018	50 (28.4)	3 (7.7)	47 (34.3)	
2019	48 (27.3)	11 (28.2)	37 (27.0)	
2020	30 (17.0)	8 (20.5)	22 (16.1)	
2021	18 (10.2)	11 (28.2)	7 (5.1)	
Year of surgery				<0.001 ^‡^
2017	11 (6.3)	1 (2.6)	10 (7.3)	
2018	47 (26.7)	7 (17.9)	40 (29.2)	
2019	50 (28.4)	9 (23.1)	41 (29.9)	
2020	38 (21.6)	6 (15.4)	32 (23.4)	
2021	30 (17.0)	16 (41.0)	14 (10.2)	
Age				0.282 ^†^
Mean ± standard deviation	65.1 ± 9.8	63.6 ± 11.4	65.5 ± 9.3	
Minimum-Maximum	24.0–83.0	24.0–83.0	30.0–83.0	
Sex				0.500 ^‡^
Female	34 (19.3)	9 (23.1)	25 (18.2)	
Male	142 (80.7)	30 (76.9)	112 (81.8)	
Race/ethnicity				0.072 ^§^
White	122 (69.3)	23 (59.0)	99 (72.3)	
African-American	6 (3.4)	2 (5.1)	4 (2.9)	
Hispanic	17 (9.7)	4 (10.3)	13 (9.5)	
Asian/Pacific Islander	23 (13.1)	5 (12.8)	18 (13.1)	
Native American/Multiracial/Other/Unknown	8 (4.5)	5 (12.8)	3 (2.2)	
Body mass index				0.055 ^†^
Mean ± standard deviation	27.0 ± 5.1	25.6 ± 4.9	27.4 ± 5.2	
Smoking history				0.976 ^‡^
Yes	13 (7.4)	3 (7.7)	10 (7.3)	
Never	61 (34.7)	14 (35.9)	47 (34.3)	
Former Smoker	102 (58.0)	22 (56.4)	80 (58.4)	
Alcohol abuse				0.103 ^§^
0	161 (91.5)	33 (84.6)	128 (93.4)	
1	15 (8.5)	6 (15.4)	9 (6.6)	
Charlson *Comorbidity* Index				0.727 ^‡^
0-3	27 (15.3)	7 (17.9)	20 (14.6)	
4-6	34 (19.3)	6 (15.4)	28 (20.4)	
7+	115 (65.3)	26 (66.7)	89 (65.0)	
Neoadjuvant chemotherapy/radiation				0.075 ^§^
0	13 (7.4)	0	13 (9.5)	
1	163 (92.6)	39 (100)	124 (90.5)	
Operative duration (in minutes)				0.695 ^†^
Mean ± standard deviation	225.0 ± 70.1	221.1 ± 73.6	226.1 ± 69.4	
Cancer clinical stage				0.002 ^‡^
I	57 (32.4)	5 (12.8)	52 (38.0)	
II	41 (23.3)	7 (17.9)	34 (24.8)	
III	47 (26.7)	14 (35.9)	33 (24.1)	
IV	31 (17.6)	13 (33.3)	18 (13.1)	
Early vs. late clinical stage				<0.001 ^‡^
I/II	98 (55.7)	12 (30.8)	86 (62.8)	
III/IV	78 (44.3)	27 (69.2)	51 (37.2)	
Histology				0.866 ^‡^
Adenocarcinoma	154 (87.5)	34 (87.2)	120 (87.6)	
Squamous	20 (11.4)	5 (12.8)	15 (10.9)	
Other	2 (1.1)	0	2 (1.5)	
Adjuvant chemotherapy/radiation				<0.001 ^‡^
0	111 (63.1)	15 (38.5)	96 (70.1)	
1	65 (36.9)	24 (61.5)	41 (29.9)	
90 day mortality				0.351 ^§^
0	169 (96.0)	39 (100)	130 (94.9)	
1	7 (4.0)	0	7 (5.1)	
1 year mortality				
0	147 (83.5)	35 (89.7)	112 (81.8)	0.235 ^‡^
1	29 (16.5)	4 (10.3)	25 (18.2)	

^†^ Two-sample t-test; ^‡^ Chi-square test; ^§^ Fisher’s exact test.

**Table 2:** Multivariable logistic regression of one year mortality.

Variables	Adjusted Odds Ratio (95% Confidence Interval)	*p*-Value
Age (year)	1.02 (0.98–1.07)	0.35
Received immunotherapy	0.54 (0.17–1.73)	0.30
Received adjuvant chemoradiation	0.91 (0.37–2.24)	0.84

**Table 3:** Adverse events and treatment completion by type of immunotherapy.

Variables	Nivolumab (N = 19) ^†^	Pembrolizumab (N = 17)	Trastuzumab (N = 11)	Ipilimumab (N = 2)
Adverse event (Y)	8	7	3	2
Type of adverse event				
Colitis	2	1		
Fatigue, loss of appetite, weight loss	1	1	2	
Hepatitis	1	1		1
High LFTs	1			
Rash	2	1		1
Neuropathy	1			
Diarrhea/UTI/AKI		1		
Neutropenia		1		
Pneumonitis		1		
Decreased LVEF			1	
Dosing frequency				
q2w	12		6	
q3w	1	13	5	1
q4w	2			
q6w	1	4		1
Unknown	3			
Completed Treatment (Y) ^§^	6	1	1	1

^†^ Sum of each mediation exceeds total number of patients on immunotherapy (*n* = 39) because 9 patients received combination of medications; ^§^ Different from sum of total patients who completed treatment (*n* = 7) because some patients had multidrug regimen.

**Table 4:** Patient demographics and mortality by receipt of immunotherapy for patients with only stage II or III pathologic stage who received surgery in 2021.

Variables	Total (N = 16)	on Immunotherapy (N = 10)	Not on Immunotherapy (N = 6)	*p* Value
Surgery in 2021	16 (100)	10 (100)	6 (100)	
Age				0.735 ^†^
Mean ± standard deviation	66.8 ± 8.0	67.3 ± 9.6	65.8 ± 4.7	
Sex				0.093 ^‡^
Female	5 (31.3)	5 (50.0)	0	
Male	11 (68.8)	5 (50.0)	6 (100)	
Race/ethnicity				0.679 ^‡^
White	13 (81.3)	7 (70.0)	6 (100)	
African-American	1 (6.3)	1 (10.0)	0	
Asian/Pacific Islander	2 (12.5)	2 (20.0)	0	
Body Mass Index				0.625^†^
Mean ± standard deviation	25.9 ± 4.9	25.8 ± 6.0	26.1 ± 3.0	
Smoking history				0.412 ^‡^
Yes	0	0	0	
Never	5 (31.3)	3 (30.0)	2 (33.3)	
Former Smoker	11 (68.8)	7 (70.0)	4 (66.7)	
Alcohol abuse				0.250 ^‡^
0	13 (81.3)	7 (70.0)	6 (100)	
1	3 (18.8)	3 (30.0)	0	
Charlson Comorbidity Index				0.281 ^‡^
0–3	5 (31.3)	4 (40.0)	1 (16.7)	
4–6	1 (6.3)		1 (16.7)	
7+	10 (62.5)	6 (60.0)	4 (66.7)	
Neoadjuvant chemotherapy/radiation	16 (100)	10 (100)	6 (100)	
Operative duration (in min)				0.082 ^†^
Mean ± standard deviation	215.3 ± 85.6	179.5 ± 32.9	274.8 ± 114.9	
Histology				1.000 ^‡^
ADENOCARCINOMA	14 (87.5)	9 (90.0)	5 (83.3)	
SQUAMOUS	2 (12.5)	1 (10.0)	1 (16.7)	
Adjuvant chemotherapy/radiation				0.118 ^‡^
0	10 (62.5)	8 (80.0)	2 (33.3)	
1	6 (37.5)	2 (20.0)	4 (66.7)	
Path stage				0.622 ^‡^
II	3 (18.8)	1 (10.0)	2 (33.3)	
IIIA	4 (25.0)	3 (30.0)	1 (16.7)	
IIIB	9 (56.3)	6 (60.0)	3 (50.0)	
Resection				0.118 ^‡^
R0	12 (75.0)	9 (90.0)	3 (50.0)	
R1	4 (25.0)	1 (10.0)	3 (50.0)	
Lymph Node Yield				0.785 ^‡^
Mean ± standard deviation	17.1 ± 8.5	15.9 ± 4.8	19.0 ± 12.9	
Min-Max	4.0–42.0	8.0–23.0	4.0–42.0	
Median (IQR)	15.0 (13.0–20.5)	15.0 (13.0–19.0)	16.0 (13.0–23.0)	
Died within 30 days of surgery				0.375 ^‡^
0	15 (93.8)	10 (100)	5 (83.3)	
1	1 (6.3)	0	1 (16.7)	
Died within 90 days of surgery				0.375 ^‡^
0	15 (93.8)	10 (100)	5 (83.3)	
1	1 (6.3)	0	1 (16.7)	
Died within 1 Year of surgery				0.206 ^‡^
0	11 (68.8)	8 (80.0)	3 (50.0)	
1	5 (31.3)	2 (20.0)	3 (50.0)	
Recurrence				1.000 ^‡^
0	4 (25.0)	3 (30.0)	1 (16.7)	
1	12 (75.0)	7 (70.0)	5 (83.3)	
Disease Free Survival (in days)				0.357 ^†^
Mean ± SD	368.7 ± 300.4	434.0 ± 339.0	259.8 ± 202.6	
Min-Max	29.0–983.0	83.0–983.0	29.0–502.0	
Median (IQR)	344.5 (118.0–512.0)	344.5 (122.0–827.0)	262.0 (63.0–441.0)	

^†^ Wilcoxon Two-sample test; ^‡^ Fisher’s exact test.

## Data Availability

The data presented in this study are available on request from the corresponding author. These data are not publicly available due to privacy policies.
